# Supplementary material for: A novel role for lipoxin A4 in driving a lymph node–eye axis that controls autoimmunity to the neuroretina
Source: eLife. 2020 Mar 2;9:e51102. doi: 10.7554/eLife.51102 (PMC7064344; doi:10.7554/eLife.51102)
Supplement: Supplementary file 1. [file elife-51102-supp1.docx]

| Key Resources Table | | | | |
| --- | --- | --- | --- | --- |
| Reagent type (species) or resource | Designation | Source or reference | Identifiers | Additional information |
| Strain, strain background (*Mus musculus*) | C57BL6J;  Wildtype;  WT | The Jackson  Laboratory | Stock #  0000664;  RRID:IMSR_JAX:000664 |  |
| Strain, strain background (*Mus musculus*) | B6.129S2-*Alox5^tm1Fun^*/J; *Alox5^-/-^* | The Jackson  Laboratory | Stock #  004155; RRID:IMSR_JAX:002263 |  |
| Strain, strain background (*Mus musculus*) | B6.129P2-*Tcrb^tm1Mom^*/J; *TCRb^-/-^* | The Jackson  Laboratory | Stock #  002118;  RRID:IMSR_JAX:002117 |  |
| Strain, strain background (*Mus musculus*) | *Fpr2^-/-^* | PMID:  20107188 |  | Obtained from the lab of Asma Nusrat, University of Michigan |
| Strain, strain background (*Mus musculus*) | B10.RIII. R161H | PMID: 23810578 |  | IRBP-specific TCR transgenic mice developed in Rachel Caspi Lab |
| Strain, strain background (*Mus musculus*) | B10.RIII. R161H - CD90.1 | PMID: 30853312 |  | Crossed and maintained in Rachel Caspi Lab |
| Strain, strain background (*Mus musculus*) | B10.RIII-*H2^r^ H2-T18^b^*/(71NS)SnJ | The Jackson Laboratory | Stock #  000457;  RRID:IMSR_JAX:000457 |  |
| Commercial assay or kit | Lipoxin A4 ELISA kit | Neogen | Cat #  407010 |  |
| Peptide, recombinant protein | IRBP_651-570_ | Genscript and  Biobasic |  | Custom orders |
| Peptide, recombinant protein | IRBP_161-180_ | Genscript and  Biobasic |  | Custom Orders |
| Other | Mycobacterium Tuberculosis H37Ra | BD | Cat #  231141 |  |
| Other | Complete Freund’s Adjuvant | Sigma-Aldrich | Cat #  F881 |  |
| Other | Pertussis toxin | Sigma-Aldrich | Cat #  P7208 |  |
| Chemical compound, drug | LXA_4_ | Cayman chemicals | Cat #  90410 |  |
| Sequence-based reagent | Alox5 | Thermo Fisher Scientific | Cat #  Mm01182747_m1 |  |
| Sequence-based reagent | Alox15 | Thermo Fisher Scientific | Cat #  Mm00507789_m1 |  |
| Sequence-based reagent | Fpr2 | Thermo Fisher Scientific | Cat #  Mm00484464_s1 |  |
| Sequence-based reagent | S1pr1 | Thermo Fisher Scientific | Cat #  Mm00514644_m1 |  |
| Chemical compound, drug | PGE_2_-d4 | Cayman chemical | Cat # 314010 |  |
| Chemical compound, drug | LTB_4_-d4 | Cayman chemical | Cat #  320110 |  |
| Chemical compound, drug | 15-(S)-HETE-d8 | Cayman chemical | Cat #  334720 |  |
| Chemical compound, drug | LXA_4_-d5 | Cayman chemical | Cat #  24936 |  |
| Chemical compound, drug | DHA-d5 | Cayman chemical | Cat #  27357 |  |
| Chemical compound, drug | AA-d8 | Cayman chemical | Cat #  390010 |  |
| Chemical compound, drug | DAPI | Sigma Aldrich | Cat #  D9542 | 1:3000 |
| Chemical compound, drug | PMA | Sigma Aldrich | Cat #  P1585 |  |
| Chemical compound, drug | Ionomycin | Sigma Aldrich | Cat #  I9657 |  |
| Chemical compound, drug | Protein Transport Inhibitor (Containing Brefeldin A) | BD | Cat #  555029 |  |
| Antibody | Purified anti-mouse CD4 Antibody, Clone RM4-5 | Biolegend | Cat #  100505;  RRID:AB_312709 | 1:200 |
| Antibody | Anti-COX2 / Cyclooxygenase 2 antibody | Abcam | Cat #  Ab15191;  RRID:AB_2085144 | 1:200 |
| Antibody | Goat anti-Rabbit IgG (H+L) Cross-Adsorbed Secondary Antibody, Alexa Fluor 488 | Thermo Fisher Scientific | Cat #  A-11008;  RRID:AB_143165 | 1:200 |
| Antibody | Goat anti-Rat IgG (H+L) Cross-Adsorbed Secondary Antibody, Alexa Fluor 568 | Thermo Fisher Scientific | Cat #  A-11077;  RRID:AB_141874 | 1:200 |
| Antibody | CD4 Monoclonal Antibody (RM4-5), Super Bright 645, eBioscience™ | Thermo Fisher Scientific | Cat #  64-0042-82; RRID:AB_2662401 | 1:1000 |
| Antibody | Alexa Fluor® 700 anti-mouse CD8a Antibody  Clone 53-6.7 | Biolegend | Cat #  100730;  RRID:AB_493703 | 1:1000 |
| Antibody | F4/80 Monoclonal Antibody (BM8), PE, eBioscience™ | Thermo Fisher Scientific | Cat #  12-4801-82;  RRID:AB_465923 | 1:1000 |
| Antibody | Ly-6G Monoclonal Antibody (1A8-Ly6g), APC, eBioscience™ | Thermo Fisher Scientific | Cat #  17-9668-82; RRID:AB_2573307 | 1:1000 |
| Antibody | IFN gamma Monoclonal Antibody (XMG1.2), Alexa Fluor 488, eBioscience™ | Thermo Fisher Scientific | Cat #  53-7311-82;  RRID:AB_469932 | 1:1000 |
| Antibody | IL-17A Monoclonal Antibody (eBio17B7), PE-Cyanine7, eBioscience™ | Thermo Fisher Scientific | Cat #  25-7177-82;  RRID:AB_10732356 | 1:1000 |
| Antibody | Mouse S1P_1_/EDG-1 APC | R&D systems | Cat #  FAB7089A; RRID:AB_10971788 | 5ul/ test |
| Antibody | CD197 (CCR7) Monoclonal Antibody (3D12), APC-eFluor 780, eBioscience™ | Thermo Fisher Scientific | Cat #  47-1979-42; RRID:AB_1518794 | 1:500 |
| Antibody | BV421 Rat Anti-Mouse CD196 (CCR6)  Clone  140706 | BD | Cat #  564736; RRID:AB_2738926 | 1:500 |
| Antibody | [BD Pharmingen™ APC Hamster Anti-Mouse CD183](https://www.bdbiosciences.com/us/applications/research/t-cell-immunology/th-1-cells/surface-markers/mouse/apc-hamster-anti-mouse-cd183-cxcr3-173/p/562266)  Clone: CXCR3-173 | BD | Cat #  562266; RRID:AB_11153500 | 1:500 |
| Antibody | PerCP/Cyanine5.5 anti-mouse CD3 Antibody  Clone 17A2 | Biolegend | Cat #  100218; RRID:AB_1595492 | 1:1000 |
| Antibody | Brilliant Violet 785™ anti-mouse/human CD44 Antibody  Clone IM7 | Biolegend | Cat #  103059; RRID:AB_2571953 | 1:1000 |
| Antibody | BV510 Rat Anti-CD11b  Clone  M1/70 | BD | Cat #  562950; RRID:AB_2737913 | 1:1000 |
| Antibody | Ghost Dye™ UV 450 | Tonbo | Cat #  13-0868-T500 | 1:2000 |
| Antibody | Ghost Dye™ Red 780 | Tonbo | Cat #  13-0865-T500 | 1:2000 |
| Commercial assay or kit | nCounter® PanCancer Mouse Immune Profiling | nanoString | Cat #  XT-CSO-MIP1-12 |  |
| Commercial assay or kit | Mouse CD3+ T Cell Enrichment Column | R&D systems | Cat #  MTCC-25 |  |
| Commercial assay or kit | EasySep™ Mouse CD4+ T Cell Isolation Kit | STEMCELL  technologies | Cat #  19852 |  |
| Peptide, recombinant protein | Recombinant Mouse CCL19/MIP-3 beta Protein | R&D systems | Cat #  440-M3-025 |  |
| Peptide, recombinant protein | Recombinant Mouse CCL21/6Ckine Protein | R&D systems | Cat #  457-6C-025 |  |
| Commercial assay or kit | Seahorse XF Glycolytic Rate Assay Starter Pack | Agilent | Cat #  103710-100 |  |
| Software, algorithm | Prism 8.2.1 | GraphPad | <https://www.graphpad.com/scientific-software/prism/>; RRID:SCR_002798 |  |
| Software, algorithm | nSolver Version 4 | nanoString | <https://www.nanostring.com/products/analysis-software/nsolver>; RRID:SCR_003420 |  |
| Software, algorithm | FIJI | NIH | <https://imagej.net/Fiji>; RRID:SCR_002285 |  |
| Software, algorithm | FlowJo 10.5.3 | FlowJo, LLC | <https://www.flowjo.com/solutions/flowjo/downloads>; RRID:SCR_008520 |  |
| Software, algorithm | Seahorse Wave 2.6.1 | Agilent | <https://www.agilent.com/en/products/cell-analysis/cell-analysis-software/data-analysis/wave-desktop-2-6>; RRID:SCR_014526 |  |
